# Investigation of Exome-Wide Tumor Heterogeneity on Colorectal Tissue-Based Single Cells

**DOI:** 10.3390/ijms26020737

**Published:** 2025-01-16

**Authors:** Nikolett Szakállas, Alexandra Kalmár, Barbara Kinga Barták, Zsófia Brigitta Nagy, Gábor Valcz, Tamás Richárd Linkner, Kristóf Róbert Rada, István Takács, Béla Molnár

**Affiliations:** 1 Department of Biological Physics, Faculty of Science, Eötvös Loránd University, 1053 Budapest, Hungary; 2 Department of Internal Medicine and Oncology, Faculty of Medicine, Semmelweis University, 1085 Budapest, Hungary; alexandra.kalmar@gmail.com (A.K.); molnar.barbara.kinga@semmelweis.hu (B.K.B.); nagyzsofiab@gmail.com (Z.B.N.); tamas.linkner@3dhistech.com (T.R.L.); kristof.rada@3dhistech.com (K.R.R.); takacs.istvan@semmelweis.hu (I.T.); molnar.bela@semmelweis.hu (B.M.); 3 HUN-REN-SU Translational Extracellular Vesicle Research Group, 1117 Budapest, Hungary; valcz.gabor@semmelweis.hu

**Keywords:** single-cell, next-generation sequencing, colorectal cancer, tumor heterogeneity

## Abstract

The progression of colorectal cancer is strongly influenced by environmental and genetic conditions. One of the key factors is tumor heterogeneity which is extensively studied by cfDNA and bulk sequencing methods; however, we lack knowledge regarding its effects at the single-cell level. Motivated by this, we aimed to employ an end-to-end single-cell sequencing workflow from tissue-derived sample isolation to exome sequencing. Our main goal was to investigate the heterogeneity patterns by laser microdissecting samples from different locations of a tissue slide. Moreover, by studying healthy colon control, tumor-associated normal, and colorectal cancer tissues, we explored tissue-specific heterogeneity motifs. For completeness, we also compared the performance of the whole-exome bulk, cfDNA, and single-cell sequencing methods based on variation at the level of a single nucleotide.

## 1. Introduction

Bulk DNA and RNA sequencing techniques are suitable for exploring the tumor microenvironment of cancer tissue. Several studies [1,2,3,4] indicate that the characteristics of both cell-extrinsic (such as the immunosuppressive tumor microenvironment, including suppressor cells and macrophages) and intracellular (epigenetic alterations, cancer cell metabolism, and oncogenic signaling) can be examined in detail. Through single-cell sequencing, we can find a systematic transcriptional atlas to delineate molecular and cellular heterogeneity as well as immune infiltration. In addition, it allows us to identify novel cell lineages and unique interactions between tumor cells and the surrounding microenvironment. Moreover, single-cell studies have revealed cancer initiative and progenitor mutations in driver genes [5]. The combination of these findings with the sampling of multiregional tissue samples can give an exact picture of the tumor’s microenvironment, particularly concerning intratumoral heterogeneity [6].

As general causes of tumor heterogeneity, we can emphasize genomic instability and enhanced clonal evolution under different environmental conditions. Intratumor heterogeneity refers to the diversity between tumor cells in a single patient [7]. This can manifest as the spatial heterogeneity that describes the distribution of genetically diverse tumor subpopulations in different tumor areas and as the temporal heterogeneity dealing with dynamic variations in the genetic diversity of an individual tumor over time [8]. Intertumor heterogeneity appears as multiple patients harbor tumors of the same histological type and are believed to result from patient-specific factors, including germline genetic variations, differences in the somatic mutation profile, and environmental conditions. A significant consequence is that, despite the dramatic initial responses, almost all cancer types resist targeted therapies due to intratumoral heterogeneity [9].

The objective of tissue isolation is to focus on specific, predetermined areas of tissues—such as transitional, differentiated, and invasive regions—that will later be subjected to various molecular analyses. Several techniques can be employed for the dissection of tissue samples, including bulk scraping, manual macrodissection, and laser capture microdissection. Choosing the most appropriate method is determined by the volume of interest (microdissection or macrodissection) and the features of the future analyses executed. Macrodissection can be performed without the use of a microscope or specialized equipment, typically targeting tumors that can be clearly defined without magnification. In contrast, during tissue microdissection, a microscope and/or other specialized equipment are also involved, such as a micromanipulator for movement, a laser source for isolation, or an adhesive tool for sample collection. Microdissectors typically operate on heterogeneous/mixed tissues, as well as on small well-defined tissue regions and functional units [10]. Regarding the origin of the target samples, formalin-fixed paraffin-embedded (FFPE) block tissues and fresh-frozen (FF) biopsies can be distinguished. Both types are suitable for macro- and microdissection procedures; however, fresh frozen tissues are used due to their potential superiority in preserving DNA and the absence of deparaffinization steps.

The process of drug resistance in various cancers, arising from tumor heterogeneity, can be observed and analyzed using single-cell RNA (scRNA) and single-cell DNA (scDNA) sequencing techniques [11,12]. Single-cell sequencing methods exploit the potential of micromanipulation and microdissection techniques. The method involves amplifying either the whole genome or specific regions of interest, constructing sequencing libraries, and employing next-generation sequencing technologies. The differences between cells are even greater for RNA, as it is more vulnerable to the influence of micro- and macroenvironmental stimuli [13]. scRNAseq provides an unprecedented opportunity and has demonstrated its utility in dissecting intratumor heterogeneity at a single-cell resolution.

Our current understanding of intratumoral heterogeneity in cancers is largely derived from the analysis of bulk tumor specimens; however, most bulk tumor specimens consist of a mixture of non-malignant cells and various subpopulations of cancer cells. Since a single tissue lesion cannot adequately represent the complex metastatic nature of many cancers, the ideal approach would involve collecting biopsy samples from multiple locations within the tumor. However, this is not a patient-friendly solution. Currently, cell-free DNA (cfDNA) profiling allows the detection of more heterogeneous driver alterations in a patient relative to single-tissue biopsies [14], but the complex picture of the mutational picture of each tumor cell with high resolution remains to be explored. Expanding on the methods using single-cell sequencing and multi-region morphology-based sampling can be an informative investigational strategy. By performing biopsy sampling of multiple regions within a single lesion, the ability to determine the extent of spatial heterogeneity within an individual tumor can be achieved more easily. The molecular makeup of cancer cells that predominate at different sites can be different due to the variable influences of microenvironment-related factors and site-specific stressors.

As part of Hungary’s Oncogenome Program in 2022, Kalmár et al. performed a large-volume study focusing on the whole-exome sequencing of colon tissues and matching cfDNA samples [15]. They found that the genes that were the most frequently mutated were *APC*, *TP53*, *TTN*, and *KRAS* in CRC tissue in the Hungarian cohort analyzed. In terms of the cfDNA WES results, tumor somatic variants were found in 6/33 CRC cases. Additionally, targeted panel sequencing was carried out on a subset of cfDNA samples. This revealed somatic variants in 8 of the 12 enrolled patients and identified 12 out of 20 tumor somatic variants within the targeted regions. In contrast, WES recovered only 20% of variants in the same targeted regions from the cfDNA of these patients. These contradictory results encouraged us to perform the whole-exome single-cell sequencing of samples from the same patients that had previously undergone WES plasma and bulk sequencing. Having data from the different sequencing methods facilitated the comparison of the results.

The presence of heterogeneity in malignant tumors can induce specific resistance to therapies. Although many research articles investigated this phenomenon, our methodological resources still lack tumor morphology-based heterogeneity-targeted single-cell techniques. The combination of laser microdissection and single-cell sequencing has been applied many times in studies in neuroscience [16,17,18]; breast, ovarian, and liver cancer [19,20,21]; and endometriosis [22]. These research areas mainly focused on single-cell RNA sequencing; however, whole-exome and whole-genome single-cell DNA sequencing on laser microdissected samples is rarely applied [18,22]. In addition, only a few examples corresponding to these types of investigations were presented in paraffin-embedded or fresh frozen tissues.

Encouraged by our findings, we conducted a study on colorectal cancer samples derived from tissue, which were collected using laser ablation and analyzed through single-cell whole-exome DNA sequencing. We aimed to highlight the benefits of single-cell sequencing and multi-region sampling to demonstrate the heterogeneity observed in our single-cell sequencing approach. We sequenced and examined samples from various locations within dedicated colon tissue sections to characterize tumor heterogeneity at the single-cell level. The selection of isolated samples was based on their morphological structures. Additionally, our goal was to identify characteristics related to variation and tumor heterogeneity across different sequencing methods. To achieve this, we performed both whole-exome bulk sequencing and circulating free DNA (cfDNA) sequencing of the solid and blood samples from the same patient, respectively.

## 2. Results

Based on the generated Dragen Enrichment reports for single-cell samples, we gathered general statistics regarding the number of bases, variants, depth of coverage, and the average efficiency of read and base reference mapping. The results showed mapping efficiencies of 29.12% and 27.59% for the NEG group, 58.06% and 57.62% for the NAT group, and 54.9% and 54.63% for the CRC group, respectively. The mean coverage depths, along with their corresponding standard deviations, for the different groups were as follows: NEG: 24.84 ± 37.94; NAT: 26.37 ± 39.94; and CRC: 31.53 ± 44.91. These coverage values are summarized in Table 1.

**Figure 1:**
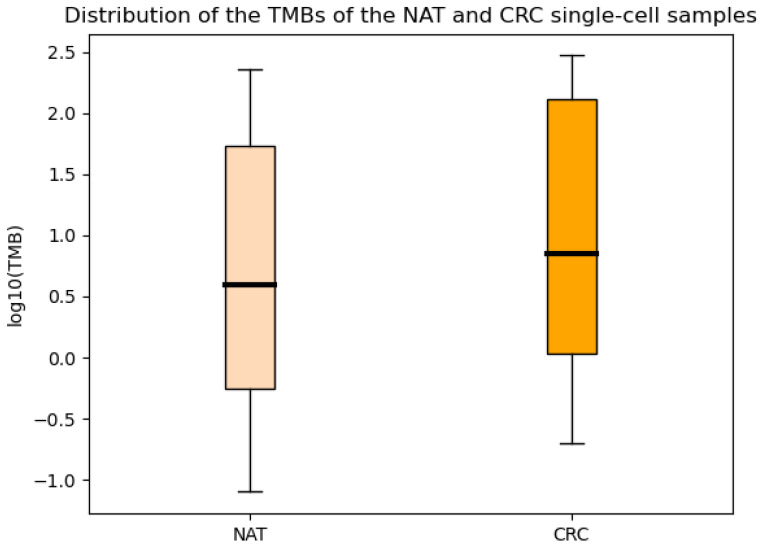
The distribution of the Tumor Mutational Burden (TMB) in the NAT and CRC samples is shown on a logarithmic scale. On average, the CRC group exhibits a higher TMB, indicating a greater number of somatic variations compared to the NAT group.

Figure 1 illustrates the distribution of tumor mutational burden (TMB) values. The boxplots regarding the TMBs of somatic variants per sample are presented on a logarithmic scale. CRC samples had a higher median value derived as 7.26 somatic alterations per megabase (Mb), while for the NAT samples, we detected 5.02 somatic variants per Mb.

**Figure 2:**
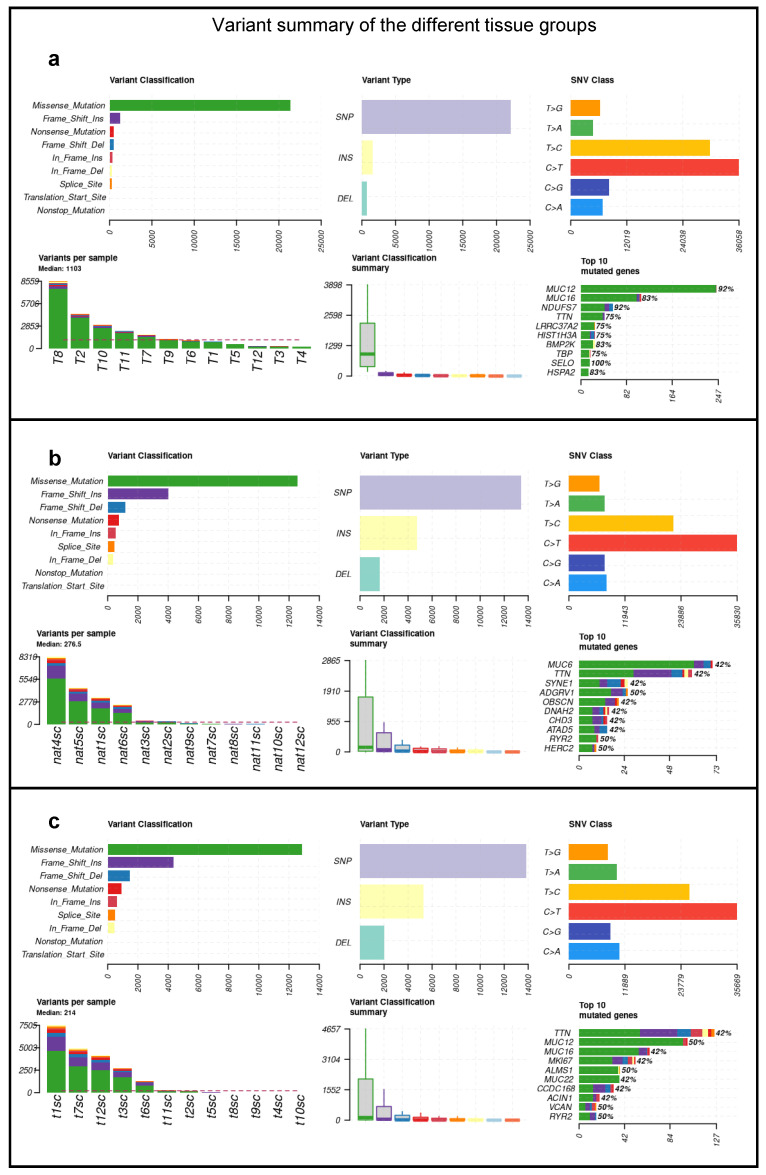
Summary plots of the (**a**) NEG, (**b**) NAT, and (**c**) CRC samples. Variant classification distribution: the *X*-axis represents the number of variants, and the *Y*-axis represents the variant type categories. Variant type plot: the *X*-axis represents the number of variants, and the *Y*-axis represents the variant type categories and SNV class plot. Variants per sample plot: the *X*-axis represents the ID of samples, and the *Y*-axis represents the number of variants. Variant classification summary: the *X*-axis represents the variant classifications, and the *Y*-axis represents the number of variants. Top 10 mutated genes: the *X*-axis represents the number of mutations, and the *Y*-axis lists the top 10 mutated genes.

The median number of mutations in the negative control samples was found to be 1103. After excluding the germline variants, the median number of mutations for the CRC and NAT groups decreased much more than for the NEG due to the applied somatic filter (Figure 2). The median number of mutations for NAT and CRC was 136 and 182, respectively, as presented in Figure 2. Detailed variant data per sample can be seen in Table A1.

**Figure 3:**
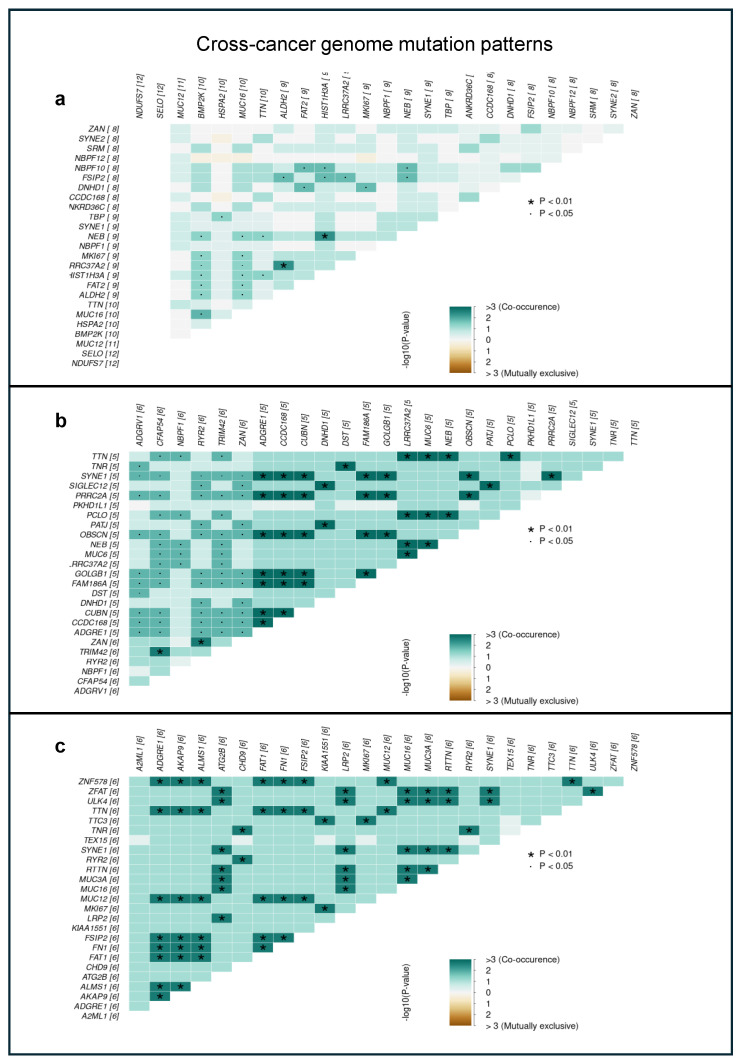
Cross−cancer genome mutation patterns may serve as a proxy to identify positive (collaboration) or negative (synthetic lethal) epistatic relationships between recurrently mutated driver genes. The epistatic relationship between two driver genes may be inferred from cross-cancer mutation patterns, whereby co-occurrence may indicate a synergistic interaction in promoting tumorigenesis. By contrast, mutually exclusive driver genes may negatively impact tumorigenesis when mutated jointly. Here, mutually exclusive and co-occurring gene pairs are presented in a triangular matrix per tissue group—(**a**) NEG, (**b**) NAT, and (**c**) CRC. Bluish-green indicates a tendency toward co-occurrence, whereas brown indicates a tendency towards mutual exclusivity. The intensity of the greenish regions corresponds to the significance of the relationship between genes, and the star symbol denotes a higher (*p* < 0.01) significance than the dot (*p* < 0.05).

To understand the relationship between genes present in the different tumor-related regions, we explored the mutually exclusive and co-occurring events by the maftools somaticInterations module. A pairwise Fisher’s exact test was performed to detect mutually exclusive and co-occurring events, and the result is presented in Figure 3. Our findings revealed that in the NEG-NAT-CRC regions, different genes play a role in the co-occurrence and exclusion, and the co-occurrence of certain genes can be observed with higher rates while their mutually exclusiveness slightly disappears in CRC. To illustrate the increase in the rate of co-occurrence, we highlight genes *RYR2* and *TNR* as examples. They co-occur significantly more times in CRC (Figure 3c, *p* < 0.01) than NAT (Figure 3b, *p* < 0.05).

### 2.1. Investigation of Tumor Heterogeneity in Single-Cell Sequenced Samples

**Figure 4:**
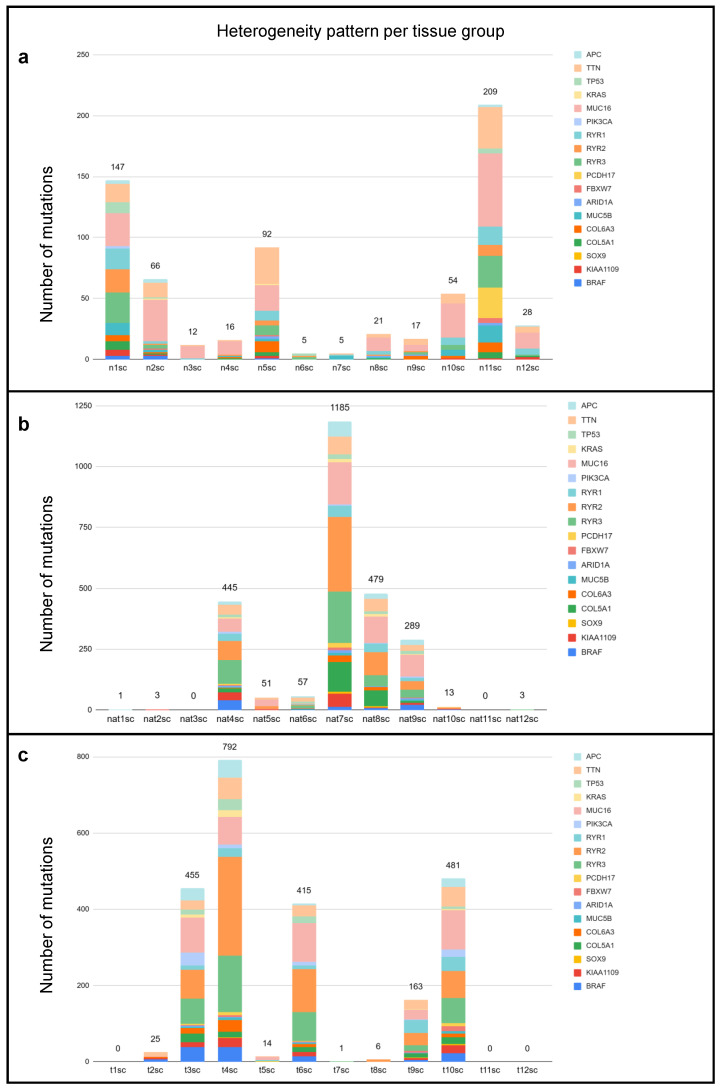
The tumor heterogeneity patterns of samples of the (**a**) NEG, (**b**) NAT, and (**c**) CRC groups. The *X*-axis represents the ID of samples, and the *Y*-axis represents the total number of mutations detected. The number of detected mutations per sample is marked above the corresponding columns, and the height of the columns is proportional to the number of detected variants. Different colors correspond to different genes. The gene-color coding is illustrated on the top-right corners of the figures.

Investigations based on single-cell heterogeneity began after identifying variants corresponding to specific groups. For the negative control, only germline variants were analyzed, while both germline and somatic variants were assessed for colorectal cancer (CRC) and normal adjacent tissue (NAT) samples. A custom Python script was used to apply certain restrictions to the variant dataset. This method focused on collecting mutations in several key genes associated with non-hypermutated colon tumors, including *APC*, *TTN*, *TP53*, *KRAS*, *MUC16*, *MUC5B*, *PIK3CA*, *BRAF*, *SOX9*, *RYR1*, *RYR2*, *RYR3*, *FBXW7*, *ARID1A*, *COL5A1*, *COL6A3*, *KIAA019*, and *PCDH17* for all locations cumulatively, and for the distinct locations one by one. The characteristics of this investigation for each sample can be seen in Figure 4. The different NEG, NAT, and CRC target regions provided similar mutation profiles but showed their uniqueness in certain aspects, for example, in the case of the mutated genes and the number of mutations, suggesting the presence of some sort of heterogeneity. Mutation profiles were derived from the different presence rates of the listed genes.

We also aimed to demonstrate the variational differences between the NAT and CRC groups from the same patient. Therefore, we created a smaller database. This database contained fewer genes and fewer samples. We introduced restrictions on the coverage values. In this examination, we only used NAT samples whose coverage was greater than 10X, and in the case of CRC, samples with coverage greater than 1X were analyzed. In the case of the CRC samples, we excluded five samples with coverage depths <1X. In the NAT group, we had to exclude six samples with coverage depths <10X. Therefore, seven CRC and six NAT samples were evaluated. The database is confined to the list of cancer-related genes that are consistently mutated in all samples as follows: *TTN*, *APC*, *KRAS*, *TP53*, *PIK3CA*, *FBXW7*, and *SOX9*. Table 2 shows that mutations on the genes *APC*, *KRAS*, *TP53*, *PIK3CA*, *FBXW7*, and *SOX9* appeared with different rates in the individual samples of groups, by 6/6 (NAT) and 6/7 (CRC), 5/6 (NAT) and 5/7 (CRC), 5/6 (NAT) and 4/7 (CRC), 3/6 (NAT) and 5/7 (CRC), 5/6 (NAT) and 5/7 (CRC), 2/6 (NAT) and 4/7 (CRC) for *APC*, *KRAS*, *TP53*, *PIK3CA*, *FBXW7*, and *SOX9*, respectively. The *TTN* gene was an exception with its unique mutation rate of 100% (6/6 (NAT) and 7/7 (CRC)).

### 2.2. Comparison of Different Input Samples

**Figure 5:**
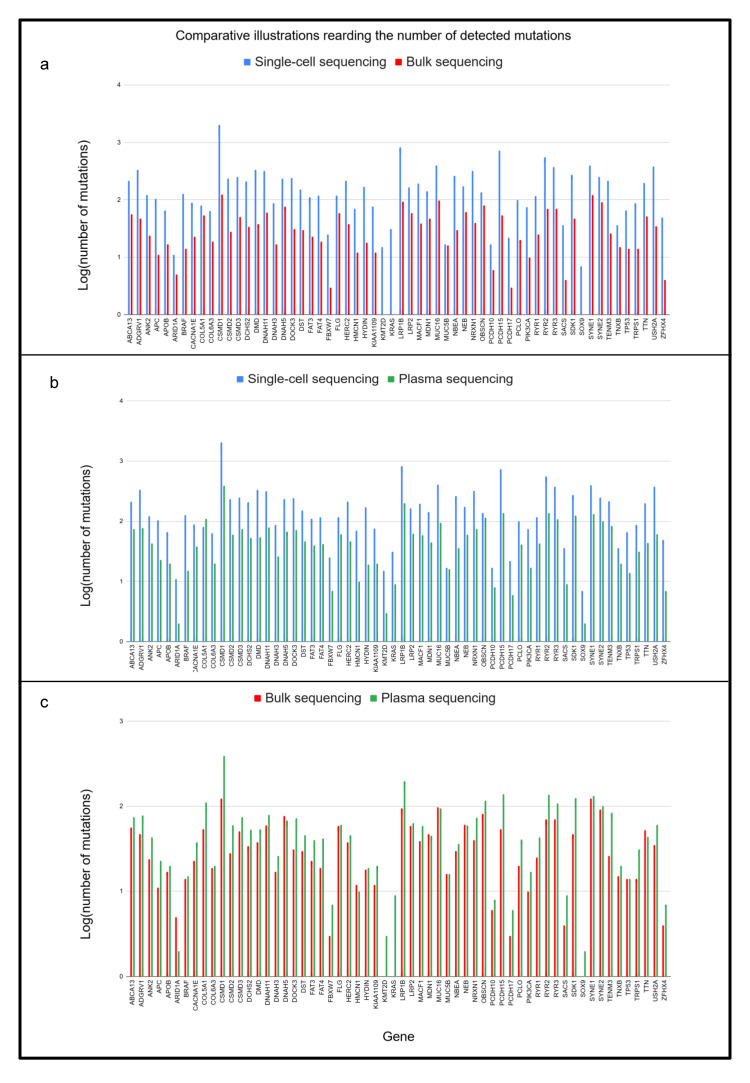
Comparative plots regarding the sequencing input samples: (**a**) single-cell vs. bulk sequencing, (**b**) cfDNA vs. bulk sequencing, and (**c**) single-cell vs. cfDNA sequencing. The *X*-axis represents the genes, and the *Y*-axis represents the number of detected mutations on a logarithmic scale. The different sequencing methods are represented by different colors, where red is associated with bulk, blue is associated with single-cell, and green is associated with cfDNA sequencing.

We also summarized the general statistics of the short-read sequencing results of the bulk exome and the matched plasma samples. According to our results, we found many more variants in the exonic regions of 12 different single-cell locations compared to a whole bulk sample (Figure 5a). The number of mutations detected in the cfDNA results fell within the bulk data range (Figure 5b) and the single-cell sequencing (Figure 5c). Moreover, we determined the mutational profiles for the 12 individual single-cell locations, which are presented in Figure 4 for groups NEG, NAT, and CRC, respectively.

**Figure 6:**
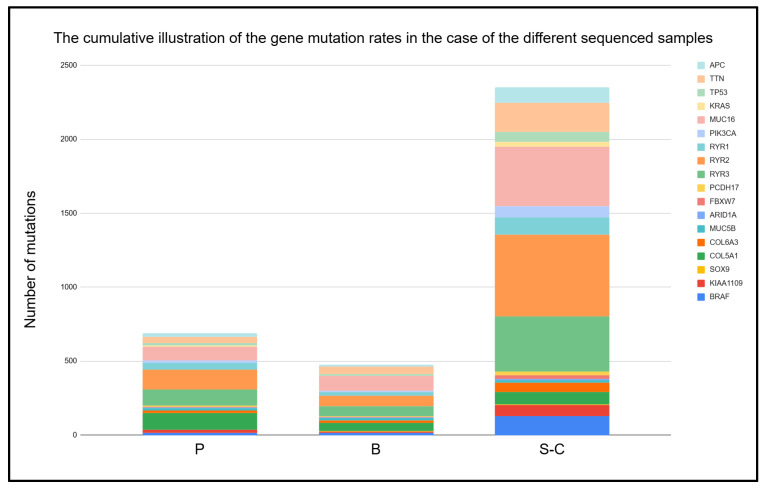
The efficiency of the different sequencing methods regarding the detection of mutations. The *X*-axis represents the different sequencing methods: (P) cfDNA, (B) bulk, and (S-C) single-cell sequencing. The *Y*-axis represents the number of detected variants. The height of the columns is proportional to the amount of detected variants. Different colors correspond to different genes. The illustrated specific genes are characteristic of non-hypermutated colon tumors: *APC*, *TTN*, *TP53*, *KRAS*, *MUC16*, *MUC5B*, *PIK3CA*, *BRAF*, *SOX9*, *RYR1*, *RYR2*, *RYR3*, *FBXW7*, *ARID1A*, *COL5A1*, *COL6A3*, *KIAA019*, and *PCDH17*. The color-coding of genes is illustrated on the top-right corners of the figure.

The outstanding performance of single-cell sequencing is presented in Figure 6, which shows that it detects many more distinct variants than the other two methods. In summarizing the plasma, bulk, and single-cell results, a ranking can be set based on the number of detected variants. Bulk sequencing provided the least detected mutations, and the cfDNA method earned second place behind single-cell sequencing. Therefore, the multi-region single-cell method can detect many more variants than whole-exome bulk and cfDNA sequencing from the same biological samples and thus provides a powerful method for investigating tumor heterogeneity. This finding is crucial for any future investigations of unique tumor-specific mutations.

To prove our findings with a clinically relevant example, based on scientific articles with a main focus on cases characterized by resistance to anti-EGFR, antiangiogenic, 5-fluorouracil (5-FU), chemo and cetuximab therapies [23,24,25,26,27], we constructed a list of genes that are mutated in patients with therapy-resistant colorectal cancer and provided the corresponding mutational attributes. These genes were the following: *APC*, *KRAS*, *TP53*, *PIK3CA*, *FBXW7*, *SMAD4*, *NRAS*, *EGFR*, *BRAF*, *RNF43*, and *ARID1A*. As we know, combined *KRAS* and *TP53* mutations are the main factors for chemotherapy resistance [26]. Furthermore, since the G12 and G13 subtypes of KRAS mutations are mainly present among colorectal patients with poor survival [28], our objective was to examine their occurrence in our sample by whole-exome sequencing. We previously confirmed by ddPCR analysis. that the sample investigated in our study does not contain the *KRAS* G12/G13 mutations. By single-cell, bulk, and cfDNA sequencing, we were also able to confirm the absence of these *KRAS* G12/G13 mutations. Encouraged by this, we performed an additional mutational analysis on the therapy-resistant mutated genes listed above and were able to detect several likely pathogenic alterations in them. Interestingly, the different methods are distinguished in the non-benign non-analogous mutation lists, which are demonstrated in Table 3. To summarize our findings, single-cell sequencing identified the most alterations (250) compared to the whole-genome results (94), consistent with our previous results. In more detail, we found 219 and 63 alterations that were revealed by single-cell and bulk sequencing, respectively, and we listed 31 mutations that were found by all methods. Further analysis of the detected mutations listed in Table A2 without a ClinVar annotation is outside of the scope of this publication.

## 3. Discussion

The primary drawback of the current scDNA- and scRNA-seq methods is the use of liquid-based samples, which do not provide any morphological information due to their bloodstream origin. To enhance the precision and efficiency of single-cell sequencing, it is necessary to obtain exact morphological information, which is present on tissue slides, and thus, we can select the specific areas of interest. This can be achieved by punching tissue blocks (PTB) or laser capture microdissection (LCM) methods. Studies have shown that LCM is more favorable for analyzing and comparing morphologically distinct patterns, including single cells or clusters of fewer than five cancer cells ahead of the invasive front [22]. The combination of LCM and two-dimensional gel electrophoresis reveals the proteomic heterogeneity in cancer surgical specimens. By distinguishing tumor cells according to tissue localization, protein spots with different intensities were observed in tumor cell groups compared to normal epithelial cells [29].

Tissue heterogeneity complicates the identification of tumor markers, and the results of the proteomic analysis of the entire tissue may be considered controversial. LCM can overcome this problem by isolating individual tumor cells. However, the small number of cells obtained by LCM severely limits the required proteome coverage and biomarker discovery potential that can be achieved using conventional proteomics platforms [30]. This disadvantage can be eliminated by the capture of multiregion tissue from the same slide as we have demonstrated in our study.

The depth of the coverage of the sequencing is an important metric for measuring the quality of variant calls. In our study, the mean coverage depths and their standard deviations per group were derived as NEG: 24.84 ± 37.94; NAT: 26.37 ± 39.94; and CRC: 31.53 ± 44.91. Based on the detailed data of Table 1, it can also be considered that in addition to the deeper coverage values (≥30X), there were many inadequate values (<10X). We can consider excluding data with low coverage; however, as we dealt with the data of individual groups collectively in most of our investigations, we kept the information corresponding to them. In the cases illustrated in Figure 4, the results of individual rare-cell locations showed that samples with low coverage gave different mutational profiles than those with higher coverage. Encouraged by this, we performed a trial to exclude data with low coverage to prove that they do not significantly influence our collective results and that they only have a slight effect. The data of this examination are presented in Table 2. However, the consequence of exclusion was that the number of samples was different between the two groups; therefore, we performed simple normalization, that is, we normalized the data derived from seven samples to that of six samples. This is denoted by subscript 6 in the summation in the last row. Normalized values showed that a higher total number of mutations can be observed in the case of NAT samples compared to CRC, even in samples with a higher coverage by the collective results.

Variants with a higher occurrence rate were detected in the NEG group assigned to the reference genome. This can be explained by the fact that the germline variant calling for this group listed all deviations from the known reference, including every individual-specific benign mutation; theoretically, the presence of the well-known somatic variations is not allowed (and this does not precisely exclude their existence). As discussed in the study of H. Lee-Six et al. [31], colorectal neoplastic changes can occur in morphologically normal tissue, and their incidence tends to increase with age. Their higher occurrence involves an increased mutational burden in normal colorectal scripts with a range of 1500–15,000 alterations per Mb. Hence, the investigation of negative cancer-free tissue biopsies allows us to gain insight into the different aspects of the earliest stages of the clonal evolution of colorectal cancers, namely, the range of mutational processes, the frequency of driver mutations, and the clonal dynamics of colonic stem cells. Furthermore, another non-scientific but relatable explanation for the increased number of mutations in NEG compared to NAT and CRC can be the ‘doubled’ amount of the input NEG sample.

**Figure A1:**
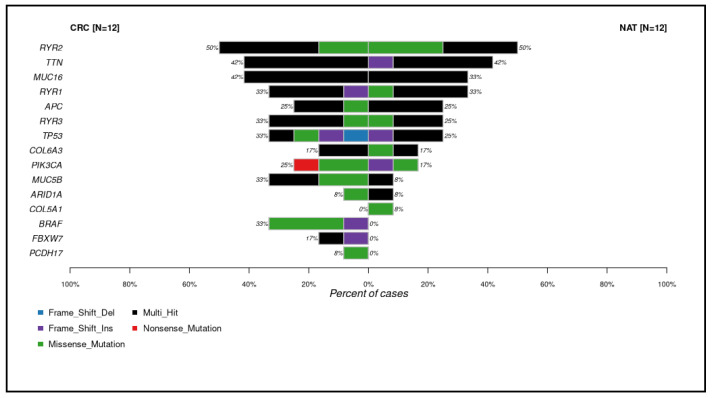
The comparative oncomutational plot of the NAT and CRC groups. The X-axis represents the occurence rate of the mutations of the genes, and the Y-axis represents the examined genes. The left-side of the figure corresponds to the CRC group, and the right-side illustrates the findings regarding the NAT group.

In Figure 5b,c, the heterogeneity patterns seem very similar for the two groups, but a deeper analysis reveals that the patterns became more diverse per sample in the case of NAT samples. In the image of the computational pattern (Figure A1, we can also observe several differences between the tissue groups. Oncogenic mutations in CRC are represented with higher rates than in NAT, especially including the genes *BRAF*, *FBXW7*, and *PCDH17*. Their mutation rates are zero in the NAT group, suggesting that they possibly have maintenance-like effects in tumor cells. Scientific resources have clarified that mutations in genes *BRAF*, *FBXW7*, and *PCDH17* contribute to resistance to therapy against immunotherapy with EGFR inhibitor and 5-FU chemotherapy, respectively [32,33,34]. Interestingly, our results show that mutations in *COL5A1* are present in NAT rather than in CRC samples, which can indicate some sort of preventive effect or may contrarily serve as a marker of tumor progression. Scientific literature data prove that the overexpression of *COL5A1* promotes tumor progression and metastasis and correlates with poor patient survival [35]. To strengthen our results, we performed an additional KEGG pathway analysis using the ShinyGO platform [36] including resistance to therapy indicating genes (*APC*, *KRAS*, *TP53*, *PIK3CA*, *FBXW7*, *SMAD4*, *NRAS*, *EGFR*, *BRAF*, *RNF43*, and *ARID1A*), similarly to the study by He et al. [37]. We found that many corresponding alterations are present in cancer development-related signaling pathways, such as the PI3K-AKT, Ras, Wnt, TGF-beta, p53, ErbB, mTOR, and MAPK pathways. Based on this, mutations on oncogenes *KRAS*, *BRAF*, and *NRAS*, and tumor suppressor genes *APC*, *SMAD4*, and *TP53* require more attention in the context of (single-cell) tumor heterogeneity and need to be investigated in more detail as we have in our study.

However, conventional anticancer therapies remove most cells from tumor mass. In the future, small surviving populations of therapy must also be considered as they evolve adaptive resistance strategies leading to treatment failure [38]. Several mutations in genes characterized by the resistance effect of therapy were identified by different sequencing systems, as presented in Table A2. We detected 38 and 11 mutations in *APC*, 7 and 0 in *KRAS*, 22 and 13 in *TP53*, 27 and 10 in *PIK3CA*, 14 and 3 in *FBXW7*, 13 and 0 in *SMAD4*, 3 and 1 in *NRAS*, 50 and 35 in *EGFR*, 67 and 14 in *BRAF*, 5 and 2 in *RNF43*, and 4 and 5 in *ARID1A* by single-cell and bulk sequencing, respectively. Additionally, we performed an analysis including specific alterations which were collected based on scientific resources [23,24,25,26,27], namely p.E542K, p.E545K, p.H1047R in *PIK3CA*, p.S192R, p.G465E, p.S492R, p.R451C, p.K467T in *EGFR*, p.V600E in *BRAF*, p.G659Vfs*41, p.G659Sfs*87, p.R117Pfs*42, p.R117S, p.R117C, p.R117Afs*41, p.R117Pfs*8, p.R117H, p.R117P, p.R117Pfs*41 in *RNF43*, and p.D1850Tfs*33, p.D1850Gfs*4, p.D1850G, p.Q309K, p.Q309*, p.Q309H, p.Q758Dfs*58, p.Q758*, p.Q758Rfs*75, and p.Q758Pfa*59 in *ARID1A*. From this detailed list, we detected p.R117H in *RNF43* using both single-cell and bulk sequencing. This alteration associated with a *BRAF* p.V600E mutation promotes a better prognosis in CRC patients who receive PD-1/PD-L1 inhibitors and the combination of anti-*EGFR/BRAF* therapy [23]. However, in our case, in the absence of this specific *BRAF* mutation, this better prognosis cannot be declared. Together, our findings confirmed the efficiency of multi-region single-cell assay in the detection and identification of therapy-resistant variations. To achieve more precise results, our assay needs methodological improvements, especially in the case of the coverage values.

In summary, the detailed analysis of genes, variants, and heterogeneity in colorectal cancer (CRC) and the neighboring normal adjacent tissue (NAT) samples leads to several conclusions. Normal sites associated with tumor regions should not be mistakenly classified as normal controls or tumor-free areas. Instead, they represent a mixture of hereditary and somatic alterations. One possible explanation for this observation is that NAT may function as a transition site between tumors and cancer-negative regions, as previously proposed by Aran et al. [39]. Furthermore, our interpretation of NAT regions is supported by the research conducted by Kim et al. [40], who found that NAT exhibits a greater number of differentially expressed genes (DEGs) compared to tumors and demonstrated better prognostic abilities. To fully understand the mutational landscape, more detailed investigations are necessary. However, it is likely that NAT contains both progenitor and inhibitor mutations, suggesting that the complexity of these interactions should not be underestimated.

As demonstrated in the paragraph discussing the sequencing coverage of our samples, the main limitation of this study is the non-equal distribution of the coverage values. Insufficient coverage data can arise due to the nature of the applied sample source, as the diameter of the collected samples was in the range of micrometers, indicating proportionally less DNA and high intersample variations. Targeted sequencing applications could resolve coverage issues independently of the sample size and origin. Our main goal here was to explore the possibility of single-cell sequencing on tissue samples and to demonstrate investigations related to heterogeneity. Another limitation of this work is the lack of characteristics related to variant and tumor heterogeneity between different sequencing methods. As it falls outside the scope of the present study, we seek opportunities to fill this gap in a future publication.

## 4. Materials and Methods

### 4.1. Clinical Samples

All samples were obtained after written informed consent forms from untreated patients were signed. Colonic specimens were collected during surgery from tumors and histologically normal adjacent tissue (NAT) at the 1st Department of Surgery, Semmelweis University, Budapest, Hungary. The samples were then stored at −80 °C until use. In addition, tissue samples from the same locations were immediately fixed in buffered formalin and experienced pathologists established the histological diagnoses. The study was carried out according to the Declaration of Helsinki and was approved by the local ethics committee and government authorities (Regional and Institutional Committee on Science and Research Ethics (ETT TUKEB); No.: 14383-2/2017/EKU Semmelweis University, Budapest, Hungary).

Fresh frozen colorectal tissue samples were embedded in Tissue-Tek^®^ O.C.T.^TM^ Compound (Sakura Finetek, Torrance, CA, USA). Cryosections (15 μm) were prepared and mounted on MMI MembraneSlides (Molecular Machines & Industries GmbH, Eching, Germany). Cryo Slides were then stored at −80 °C for later use. Hematoxylin-eosin staining was performed to visualize the tissue morphology. For dehydration, slides were first dipped in 90% ethanol for 10 min, then in 100% ethanol for 10 min, and finally in xylene for 5 min.

Single-cell samples were cut from a membrane frame slide by laser microdissection (Laser Capture Microdissection, Molecular Machines & Industries GmbH). Target areas with a diameter of 30–40 μm and a height of approximately 20–30 μm were collected. The height was derived from the thickness of the membrane slide (4–6 μm) and the thickness of the tissue section (6–20 μm). The samples were selected based on their morphological structure located at random sites of the histological sections. Each group contained circles from 12 different regions of the corresponding slide. Sample collection was performed using 0.2 mL MMI Diffuser caps (Molecular Machines & Industries GmbH). Overall, we had 12-12 NAT and colorectal cancer (CRC), and 12(x2: the size of the pieces was doubled to two neighboring pieces to ensure a sufficient sample amount; however, in later steps, these pairs were treated as single samples) cancer-negative healthy samples (NEG). We attempted to perform laser microdissection on samples that met specific morphological criteria. For the NEG group, any areas with signs of inflammation were strictly excluded. In the case of the NAT group, we selected areas that were closest to tumorous sites but were confirmed by a pathologist to be non-cancerous. Since our examination focuses on colorectal cancer tissues, we assumed the absence of other cancers. Slides were observed using an inverted microscope (Fully Motorized and Automated Inverted Microscope IX83, Olympus Life Science, Waltham, MA, USA) before and after dissection.

### 4.2. Single-Cell DNA Extraction, Library Preparation, and Next-Generation Sequencing

DNA extraction and whole-genome amplification were performed by the REPLI-g Single Cell Kit (Qiagen GmbH, San Diego, CA, USA) according to the manufacturer’s instructions. Briefly, as a first step, cells collected by laser microdissection were lysed and DNA was denatured at 65 °C for 30 min. After adding a neutralization buffer that stops denaturation, whole-genome amplification was performed by REPLI-g sc-DNA Polymerase with incubation at 30 °C for 2 h and enzyme inactivation at 65 °C for 3 min. Following quantity and quality control by the Qubit HS dsDNA kit on the Qubit 1.0 fluorometer (Thermo Fisher Scientific, Waltham, MA, USA) and high sensitivity DNA chip on Bioanalyzer 2100 microcapillary electrophoresis system (Agilent Technologies, Santa Clara, CA, USA), 200–1000 ng of amplified DNA were further processed. For the library preparation, the QIAseq FX Single Cell DNA Library kit (Qiagen GmbH) was applied. Enzymatic fragmentation was performed with FX Enhancer for 4 °C 1 min (pre-chilled thermocycler), 32 °C for 15 min, 65 °C for 30 min, and 4 °C hold. After the adapter ligation, clean-up was applied with 0.8× Agencourt AMPure XP (Beckman Coulter, Indianapolis, IN, USA) and then with 1× AMPure XP beads. PCR-based library amplification was used with QIASeq HiFi Mastermix (Qiagen GmbH) under the following thermocycling conditions: 98 °C for 2 min, 6 cycles of 98 °C for 20 s, 60 °C for 30 s, 72 °C for 30 s, followed by a final extension of 72 °C for 1 min. Post-amplification clean-up was performed with 1× AMPure XP Beads. Quality was verified with BioAnalyzer 2100 and quantity was assessed by QIAseq^TM^ Library Quant Assay Kit (Qiagen GmbH) according to the manufacturer’s instructions.

Whole-exome capture was completed using the QIASeq Human Exome Kit (Qiagen GmbH) with 200 ng input DNA per sample. For hybridization capture, 6-6 indexed libraries were pooled and the reaction was carried out according to the manufacturer’s instructions. Following the binding of the hybridized targets to streptavidin beads, washing steps were completed and postcapture amplification was carried out with the Illumina Library Amplification Post Hybrid Capture PCR Mix and Primer Mix Illumina Library Amplification with the following thermocycling conditions: 98 °C for 2 min, 7 cycles of 98 °C for 20 s, 60 °C for 30 s, and 72 °C for 30 s, then 72 °C for 1 min. Clean-up was completed with 1.5× AMPure XP Beads. The quality of the library was again evaluated with BioAnalyzer 2100, and the exact amount of libraries was quantified with the QIAseq^TM^ Library Quant Assay Kit (Qiagen GmbH) according to the manufacturer’s instructions. A 4-4 nm library pool was pooled together, and this sample was further prepared according to the Denature and Dilute Libraries Guide (Illumina Inc., San Diego, CA, USA). Finally, 12 samples per run were examined.

Paired-end next generation sequencing was performed using the NextSeq High Output Kit on a NextSeq 500/550 Instrument (Illumina Inc.) with 149 cycles for Read 1 and Read 2 and 10 cycles for Index 1 and 2 (corresponding to the QIAseq UDI Y-Adapters (Qiagen Gmbh) protocol).

### 4.3. Bioinformatic Analyses

The same short-read sequencing bioinformatic analysis was performed on single-cell, whole-exome bulk, and cfDNA sequencing data. The results of whole-exome bulk and plasma sequenced samples were available from the study of Kalmár et al. [15]. Briefly, we would like to summarize the mutual evaluation steps. The demultiplexing and FASTQ file generation was performed using the Illumina BaseSpace interface (Illumina Inc.). Next, we used the FastQC and MultiQC tools to assess the quality of sequencing reads. The raw sequence reads were aligned with the Human Reference Genome GRCh38. SNP and short indel germline and somatic variants were called and determined by the Dragen Germline Variant Caller v. 4.2.4 (Illumina Inc.) on the NEG, CRC, and NAT samples. Dragen Somatic Variant Caller v. 4.2.7 (Illumina Inc.) was run in tumor-normal mode for the CRC and NAT samples.

The variant call.vcf files were annotated using the SnpEff eff variant annotation tool on the Galaxy website [41], and the mutation annotation.maf files were generated by the package vcf2maf [42] and the Ensembl Variant Effect Predictor (VEP) release with version 102 [43]. The clinical impact of the variants was evaluated according to the ClinVar [44] database. Variant characteristics were summarized using the maftools [45] ‘plotmafsummary’ tool. The summary plots for the CRC and NAT groups include only the somatic mutations. The tumor mutation burden (TMB) values were calculated using the “tmb” function of the maftools program package as the number of non-silent mutations per mega base (Mb) for each data group. The target capture size was set to 37 Mb according to the exome sequencing kit used for our samples. An oncoplot and the landscape of somatic interactions were made by the oncoplot and somaticInteractions modules of maftools, respectively. To perform the KEGG pathway analysis of therapy-resistant genes, we used the ShinyGO platform [36].

After summarizing the results of the different sequencing methods, several analyses were performed in the Python and R program languages, and a cumulative data table per method was created to make data illustration easier. The heterogeneity-related bar plots were generated in Microsoft Excel based on the cumulative table, where the columns represented the different locations and the rows indicated for the gene mutations. The TMB boxplots were created using the matplotlib package [46] using the pyplot.boxplot tool.

## 5. Conclusions

In conclusion, we can declare that the different sequencing methods detect mutations on different scales. According to our findings regarding the investigated sequencing methods, the single-cell sequencing of tissue samples has a superior performance compared to the others in terms of heterogeneity- and resistance-based therapy investigations. With regard to the results demonstrated, we can conclude that sequencing different sample pieces from various locations rather than analyzing the whole bulk or cfDNA samples demonstrates better potential, especially when the goal is to present region-specific mutational patterns. Although we completed single-cell sequencing using a short-read next-generation sequencing instrument, the possibility of achieving much more compact results with a long-read third-generation device still holds and needs to be investigated.

## Figures and Tables

**Table 1:** Mean coverage depth values for individual samples with columns representing sample IDs and rows indicating different tissue groups. Each row–column intersection reflects the unique coverage values.

	1	2	3	4	5	6	7	8	9	10	11	12	Mean	STD
NEG	8.2	25.1	2.5	4.2	0.5	10.9	76.6	126	8.9	19.2	14.3	1.7	24.8	37.9
NAT	37.2	11.4	13.5	125.5	85.3	33.7	0.8	0.7	5.6	0.9	1.6	0.2	26.4	39.9
CRC	141.2	12.6	66	0.3	1	14.1	62	0.8	0.4	0.1	5	74.8	31.5	44.9

**Table 2:** Heterogeneity mutation data of single-cell samples over genes *TTN*, *APC*, *KRAS*, *TP53*, *PIK3CA*, *FBXW7*, and *SOX9*. Only samples with adequate coverage (for CRC >1X, for NAT >10X) were included. This database presents the occurrence and number of mutations on the specific genes per sample. The last row of the table shows the total number of mutations for all samples. In columns where the number 6 appears next to the summation sign, the total number of mutations has been normalized based on data from six samples. This normalization was necessary due to the unequal number of samples in the different groups.

NAT				CRC			
Gene	Number of Mutations	Samples Containing the Mutated Gene	Occurence Rate of the Mutated Genes	Gene	Number of Mutations	Samples Containing the Mutated Gene	Occurence Rate of the Mutated Genes
*TTN*	174	6/6	100%	*TTN*	197	7/7	100%
*APC*	137	6/6	100%	*APC*	197	5/7	71%
*KRAS*	73	5/6	83%	*KRAS*	45	5/7	71%
*TP53*	62	5/6	83%	*TP53*	66	4/7	57%
*PIK3CA*	10	3/6	50%	*PIK3CA*	38	5/7	71%
*FBXW7*	14	5/6	83%	*FBXW7*	25	5/7	71%
*SOX9*	7	2/6	33%	*SOX9*	7	4/7	57%
∑ = 477		$\sum_{6}$ = 477		∑ = 498		$\sum_{6}$ = 427	

**Table 3:** The non-benign mutations identified by the single-cell and bulk sequencing assays. The variant results were annotated using the ClinVar database. This table lists only those mutations that have previously been reported with clinical significance.

Single-Cell Sequencing			
**Gene**	**Sequence Variation**	**dbSNP ID**	**Mutation Classification**
*BRAF*	c.1763T>C	rs1562954580	uncertain significance
*BRAF*	c.1141-1111G>A	rs373442098	conflicting classifications of pathogenicity
*TP53*	c.424C>T	rs1597371187	uncertain significance
*SMAD4*	c.692dupG	rs377767334	pathogenic
*APC*	c.559+37C>A	rs1554084977	uncertain significance
*APC*	c.560-84T>C	rs1561605775	uncertain significance
**Bulk Sequencing**			
**Gene**	**Sequence Variation**	**dbSNP ID**	**Mutation Classification**
*APC*	c.136-1428A>C	rs2464807	other
*APC*	c.2413C>T	rs587779783	pathogenic
*APC*	c.4666dupA	rs587783031	pathogenic
*EGFR*	c.3368C>T	rs775317295	uncertain significance
*TP53*	c.877-1G>A	rs587782272	pathogenic/likely pathogenic

## Data Availability

Due to privacy issues, we do not provide full data availability regarding the single-cell sequencing part of this publication. The data are used according with the consent provided by the participants without compromising their anonymity. Upon request, we can provide data for peer review purposes. As we used the results of the bulk and cfDNA sequencing of a previous publication, these are available at https://cbioportal.vo.elte.hu/cbioportal (accessed on 19 September 2022).

## Appendix A

**Table A1:** Summary of coverage, tmb, fragment length, and the number of different mutation types of the NEG, NAT, and CRC data. The variant results of the NAT and CRC groups are expanded by the data of somatic alterations.

				Germline			Somatic		
**ID**	**Mean Region Coverage Depth**	**TMB**	**Median Fragment Length**	**SNP**	**Deletion**	**Insertion**	**SNP**	**Deletion**	**Insertion**
NEG1	8.2	22.1	194	14,775	6	223			
NEG2	25.1	95.38	198	48,084	32	503			
NEG3	2.5	7.74	170	4280	16	422			
NEG4	4.2	6.04	179	5268	12	354			
NEG5	0.5	18.56	180	102	0	6			
NEG6	10.9	24.82	176	7490	188	340			
NEG7	76.6	39.8	191	60,524	1523	2158			
NEG8	126	178.72	191	183,465	137	3068			
NEG9	8.9	27.8	178	14,895	6	227			
NEG10	19.2	66.26	202	37,999	16	529			
NEG11	14.3	8.02	206	28,891	15	692			
NEG12	1.7	52.3	228	4359	0	62			
NAT1	37.2	73.94	264	92,880	101	1305	55,002	3501	5355
NAT2	11.4	8.54	227	14,040	12	141	8906	636	1169
NAT3	13.5	8.16	217	18,970	41	338	11,348	869	1178
NAT4	125.5	226.52	198	261,094	54	110	137,773	7286	10,311
NAT5	85.3	108.54	164	134,821	20	86	73,450	3753	5428
NAT6	33.7	48.28	220	57,992	87	714	35,854	2470	3594
NAT7	0.8	1.78	268	1892	12	87	1125	94	169
NAT8	0.7	0.56	214	1518	2	19	979	75	126
NAT9	5.6	1.88	205	3387	10	109	2196	164	266
NAT10	0.9	0.18	224	970	3	25	717	47	62
NAT11	1.6	0.54	239	950	2	24	630	47	80
NAT12	0.2	0.08	257	230	0	5	159	8	22
T1	141.2	294.7	241	232,133	148	1516	136,632	1355	9003
T2	12.6	5.68	255	5510	16	168	3743	126	494
T3	66	118.04	212	103,007	66	685	61,950	641	3810
T4	0.3	0.64	266	270	3	7	220	4	42
T5	1	0.86	255	665	1	23	505	15	53
T6	14.1	51.66	259	24,802	48	444	15,029	349	1545
T7	62	205.2	253	137,039	169	1724	82,054	1363	6703
T8	0.8	1.16	259	1286	1	46	679	27	111
T9	0.4	1.38	244	385	0	23	236	4	41
T10	0.1	0.2	305	110	0	5	68	4	18
T11	5	8.84	260	6608	14	126	3854	126	503
T12	74.8	176.26	231	93,550	120	1121	57,145	1161	5684

**Table A2:** Mutations on genes *APC*, *ARID1A*, *BRAF*, *EGFR*, *FBXW7*, *KRAS*, *NRAS*, *PIK3CA*, *RNF43*, *SMAD4*, and *TP53* contributing to therapy resistance. The table is divided to 3 parts, where the detected mutations are listed by single-cell, bulk, and both sequencing methods. The common mutations are listed independently in the last part, and they are not included in the lists of individual methods.

Gene	Sequence Variation	dbSNP ID	Mutation Classification
Mutations Detected by Single-Cell Sequencing Only			
*APC*	n.112707592dupG		
*APC*	c.135+5252G>C		
*APC*	c.135+5253A>T		
*APC*	c.135+5254G>C		
*APC*	c.136-230C>A	rs2464805	benign
*APC*	c.221-291C>A		
*APC*	c.4853dupT		
*APC*	c.6172G>T		
*APC*	c.*433T>A		
*APC*	c.*1958_*1959insTTAC		
*APC*	c.*1965T>C		
*APC*	c.*2220_*2221ins		
*APC*	c.934-8_934-7ins	rs1561535860	likely benign
*APC*	c.934-4dupA		
*APC*	c.5613T>C		
*APC*	c.*267_*273delCCATCCC		
*APC*	c.*281_*283delTTT	rs42427	benign
*APC*	c.*285A>G	rs866006	benign
*APC*	c.*1098T>C		benign
*APC*	c.*1556C>G		
*APC*	c.560-12T>C		
*APC*	c.1881-762G>A	rs41116	benign
*APC*	c.*413_*414dupAA	rs2289484	benign
*APC*	c.559+37C>A	rs1554084977	uncertain significance
*APC*	c.559+223C>T	rs41115	benign
*APC*	c.627_628insAGAAGATGAA	rs1580673845	benign
*APC*	c.628_628+1ins	rs2229995	benign
*ARID1A*	c.*421delA		
*ARID1A*	c.*724C>A		
*ARID1A*	c.2295-161delT		
*ARID1A*	c.2496-130_2496-128delAAA		
**Mutations Detected by Single-Cell Sequencing Only**			
*BRAF*	c.1763T>C	rs1562954580	uncertain significance
*BRAF*	c.1695-940C>A		
*BRAF*	c.1695-1205G>A		
*BRAF*	c.1695-5750C>G		
*BRAF*	c.1694+8566G>A		
*BRAF*	c.1694+3374T>G		
*BRAF*	c.1694+3266A>G		
*BRAF*	c.1694+2940C>T		
*BRAF*	c.1140+3214G>A		
*BRAF*	c.1140+2430C>T		
*BRAF*	c.1140+2069A>G		
*BRAF*	c.1140+1915G>T		
*BRAF*	c.1140+1665dupG		
*BRAF*	c.981-2296A>G		
*BRAF*	c.980+2576T>A		
*BRAF*	c.980+1801_980+1802delAA		
*BRAF*	c.241-198G>C		
*BRAF*	c.981-356dupA		
*BRAF*	c.981-1080_981-1042del		
*BRAF*	c.589G>A		
*BRAF*	c.243C>A		
*BRAF*	c.984-1276C>T		
*BRAF*	c.1315-470C>T		
*BRAF*	c.1315-479A>C		
*BRAF*	c.1315-482A>T		
*BRAF*	c.451-200C>T		
*BRAF*	c.1251+48_1251+49delGA		
*BRAF*	c.1178-1544T>C		
*BRAF*	c.1178-1548A>G		
*BRAF*	c.1178-1551C>T		
*BRAF*	c.1178-1557C>T		
*BRAF*	c.981-2446G>C		
*BRAF*	c.160delC		
*BRAF*	c.1394T>C		
*BRAF*	c.328dupT		
*BRAF*	c.1804-85C>A		
*BRAF*	c.2138C>T		
*BRAF*	c.814-174G>A		
*BRAF*	c.814-77_814-76insAATA		
*BRAF*	c.1059+170C>A		
*BRAF*	c.1737A>G		
*BRAF*	c.1141-1044C>T		
*BRAF*	c.1141-1097A>G		
*BRAF*	c.1141-1111G>A	rs373442098	uncertain significance
*BRAF*	c.1141-1637_1141-1636delTT		
*BRAF*	c.1140+633C>G		
*BRAF*	c.1140+610_1140+615delAGCTAT		
*BRAF*	c.861-75dupT		
*BRAF*	c.860+457delC		
*BRAF*	c.112-5717G>T		
*BRAF*	c.112-5732A>G		
*BRAF*	c.112-5778A>G		
*BRAF*	c.112-6770_112-6769insA		
*BRAF*	c.112-7499A>G		
*BRAF*	c.112-7501A>T		
*BRAF*	c.112-7503C>T		
*BRAF*	c.*274+536A>G		
*BRAF*	c.983+1398T>C		
*BRAF*	c.983+1236_983+1237insCAAGAGGT		
*BRAF*	c.983+1233_983+1234delTT		
*BRAF*	c.983+1232T>C		
*BRAF*	c.983+1186G>A		
*BRAF*	c.876+630A>G		
*EGFR*	c.88+48720_88+48721delTG		
*EGFR*	c.322A>T		
*EGFR*	c.2469+5015G>A		
*EGFR*	c.2625+13C>G		
*EGFR*	c.2947-203G>A		
**Mutations Detected by Single-Cell Sequencing Only**			
*EGFR*	c.3272-1104_3272-1092del		
*EGFR*	c.3272-1071delG		
*EGFR*	c.3272-1068delA		
*EGFR*	c.3272-1064_3272-1063insAAAA		
*EGFR*	c.3272-438T>C		
*EGFR*	c.3272-408C>A		
*EGFR*	c.*932dupA		
*EGFR*	c.3271+191T>A		
*EGFR*	c.3271+191T>G		
*EGFR*	c.3271+1166C>A		
*EGFR*	c.3272-1115_3272-1099del		
*EGFR*	c.*781G>C		
*EGFR*	c.*1151dupC		
*EGFR*	c.*1382dupT		
*EGFR*	c.*1957G>A		
*EGFR*	c.1695-2105C>G		
*EGFR*	c.1741+165A>G	rs10228436	benign
*EGFR*	c.1695-1134A>G	rs2227984	benign
*EGFR*	c.2469+108delG		
*EGFR*	c.3271+188T>G		
*EGFR*	c.3271+282T>A	rs2075110	benign
*EGFR*	c.1721A>G		
*EGFR*	c.1314+556_1314+557dupTT	rs10241451	benign
*EGFR*	c.1314+394A>G		
*EGFR*	c.1314+256G>A		
*EGFR*	c.1178-443A>G		
*EGFR*	c.1178-648G>A		
*EGFR*	c.3271+809G>A	rs2072454	benign
*EGFR*	c.3271+976G>C		
*EGFR*	c.3272-611_3272-608delTACA		
*EGFR*	n.140724136T>C	rs2075109	benign
*EGFR*	n.140726106G>C		
*EGFR*	c.2128-5dupT		
*EGFR*	c.2128-27C>T		benign
*EGFR*	c.1993-90G>T	rs2227983	benign
*EGFR*	c.1993-93A>C	rs2227984	benign
*EGFR*	c.1742-352C>G	rs2241055	benign
*EGFR*	c.1742-353C>G		
*EGFR*	c.1741+318A>G		
*EGFR*	c.1695-53G>A		
*EGFR*	c.1314+557dupT		
*FBXW7*	c.2001dupG		
*FBXW7*	c.*1125delT		
*FBXW7*	c.*1111A>C		
*FBXW7*	c.986-147A>C		
*FBXW7*	c.1728_1729insAAACAAC		
*FBXW7*	c.1727_1728ins		
*FBXW7*	c.1721_1722ins		
*FBXW7*	c.1716_1717ins		
*FBXW7*	c.933+18A>G		
*FBXW7*	c.934-191A>T		
*FBXW7*	c.934-15_934-14delTC		
*FBXW7*	c.934-12_934-11insAC		
*KRAS*	c.876+408_876+409dupTT		
*KRAS*	c.556-72_556-71delAA		
*KRAS*	c.257C>T		
*KRAS*	c.1308+459A>C		
*KRAS*	c.1308+468C>T		
*KRAS*	c.1308+472C>T		
*KRAS*	c.1308+473A>G		
*NRAS*	c.-2070dupT		
*NRAS*	c.1251+52A>T	rs61758221	benign
*PIK3CA*	c.*3631T>C		
*PIK3CA*	c.*1606delA		
*PIK3CA*	c.1251+54A>C		
*PIK3CA*	c.1251+57G>C		
*PIK3CA*	c.1251+58C>A		
*PIK3CA*	c.*355G>T		
*PIK3CA*	c.*480C>A		
**Mutations Detected by Single-Cell Sequencing Only**			
*PIK3CA*	c.*488_*490delTCC		
*PIK3CA*	c.*494G>T		
*PIK3CA*	c.1736_1737insAAAACAAA		
*PIK3CA*	c.1735G>T		
*PIK3CA*	c.1733C>A		
*PIK3CA*	c.2496-124G>T	rs7623154	benign
*PIK3CA*	c.2923A>T	rs17550640	benign
*PIK3CA*	c.2936+21dupA		
*PIK3CA*	c.*654T>G	rs3729676	benign
*PIK3CA*	c.645+173A>G		
*PIK3CA*	c.934-132delG		
*PIK3CA*	c.3381G>C		
*PIK3CA*	c.6200A>G		
*PIK3CA*	c.6633C>T		
*RNF43*	c.*6514G>A		
*RNF43*	c.451-5T>C		
*RNF43*	c.376-90A>G		
*RNF43*	c.2936+19A>G		
*SMAD4*	c.692dupG		pathogenic
*SMAD4*	c.905-1G>C		
*SMAD4*	c.1139+385A>T		
*SMAD4*	c.*5005dupT		
*SMAD4*	c.*5116C>G		
*SMAD4*	c.*5131A>G		
*SMAD4*	c.*5191C>G		
*SMAD4*	c.*5535_*5536delAC		
*SMAD4*	c.*5541_*5552del		
*SMAD4*	c.*5863_*5867delGAAAA		benign
*SMAD4*	c.*5994A>C		benign
*SMAD4*	c.*6433G>A		
*SMAD4*	c.1060-42G>T		
*TP53*	c.984-1412delT		
*TP53*	c.983+1201A>G		
*TP53*	c.556-71delA		
*TP53*	c.556-149T>A		
*TP53*	c.424C>T		
*TP53*	c.259-161_259-158delAAAA		
*TP53*	c.259-162_259-158delAAAAA		
*TP53*	c.258+123dupT		
*TP53*	c.99dupC		
*TP53*	c.-22+41_-22+48delACCTGGAG		
*TP53*	c.-145-190C>A		
*TP53*	c.-145-1184T>C		
*TP53*	c.966dupG		
*TP53*	c.582+132T>C		
*TP53*	c.1060-69G>T		
*TP53*	c.1308+477T>C		
*TP53*	c.1308+478G>C		
*TP53*	c.1308+501C>T		
*TP53*	c.1308+521_1308+574del		
*TP53*	c.*3333C>T		
**Mutations Detected by Bulk Sequencing Only**			
*APC*	c.136-1428A>C	rs2464807	other
*APC*	c.220+124C>G	rs76552546	likely benign
*APC*	c.2413C>T	rs587779783	pathogenic
*APC*	c.4666dupA	rs587783031	pathogenic
*ARID1A*	c.1920+6177G>T		
*ARID1A*	c.1921-1059A>T		
*ARID1A*	c.2252-97A>T	rs113319329	benign
*ARID1A*	c.2733-400A>G		
*ARID1A*	c.3199-95A>G	rs76490152	benign
*BRAF*	n.140726457T>C		
*BRAF*	c.*1215A>T		
*BRAF*	c.2128-16C>T	rs368721021	benign/likely benign
*BRAF*	c.1177+146G>A	rs1267632	benign
*BRAF*	c.1140+3180G>T		
**Mutations Detected by Bulk Sequencing Only**			
*BRAF*	c.505-6693T>C		
*BRAF*	c.505-9562G>A		
*BRAF*	c.504+3486T>C		
*BRAF*	c.504+142G>A		benign
*BRAF*	c.139-23483C>T		
*EGFR*	c.88+37643T>C		
*EGFR*	c.89-55393G>A		
*EGFR*	c.89-29869C>A		
*EGFR*	c.559+214G>T	rs2270427	benign
*EGFR*	c.1498+22A>T	rs1558544	benign
*EGFR*	c.1498+142C>T	rs759162	benign
*EGFR*	c.1499-177A>G	rs11536635	benign
*EGFR*	c.1880+733A>C		
*EGFR*	c.2361G>A		benign
*EGFR*	c.2469+4027T>C		
*EGFR*	c.2508C>T		benign
*EGFR*	c.2625+196A>G	rs6970262	benign
*EGFR*	c.2709T>C	rs1140475	benign
*EGFR*	c.2849-551T>G		
*EGFR*	c.3162+200_3162+201insAG	rs34723095	benign
*EGFR*	c.3272-123G>A	rs2692456	benign
*EGFR*	c.3333_3334insTTTTTTTTTTTTT		
*EGFR*	c.3337delC		
*EGFR*	c.3339_3350delGCCTCTGAACCC		
*EGFR*	c.3353C>G		
*EGFR*	c.3355C>G		
*EGFR*	c.3356C>A		
*EGFR*	c.3368C>T	rs775317295	uncertain significance
*EGFR*	c.*9367A>G		
*FBXW7*	c.*3466C>G		
*FBXW7*	c.-69-40817T>C		
*PIK3CA*	c.1059+62C>A	rs2699895	benign
*PIK3CA*	c.1060-17C>A	rs2699896	benign
*PIK3CA*	c.1145+54A>G	rs3729679	benign
*PIK3CA*	c.2016-27A>T	rs6443625	benign
*PIK3CA*	c.*5631C>T		
*PIK3CA*	c.*10339G>T		
*RNF43*	c.2057C>G	rs9652855	benign
*TP53*	c.*4020_*4049del		
*TP53*	c.*274+522T>G		
*TP53*	c.*274+31A>G		
*TP53*	c.877-1G>A	rs587782272	pathogenic
*TP53*	c.555+62A>G		benign
*TP53*	c.259-91G>A		benign
*TP53*	c.259-160_259-158delAAA		
*TP53*	c.98C>G		benign
*TP53*	c.-22+41_-21-54del		
*TP53*	c.-44+38C>G		benign
*TP53*	c.-14962_-14959dupGTTT		
**Mutations detected by both methods**			
*APC*	c.1458T>C	rs2229992	benign
*APC*	c.4479G>A	rs41115	benign
*APC*	c.5034G>A	rs42427	benign
*APC*	c.5268T>G	rs866006	benign
*APC*	c.5465T>A	rs459552	benign
*APC*	c.5880G>A	rs465899	benign
*APC*	c.7504G>A	rs2229995	benign
*BRAF*	c.2128-54_2128-51dupCTTT		
*BRAF*	c.1992+16G>C	rs3789806	benign/likely benign
*BRAF*	c.1992+14A>G		
*BRAF*	c.1929A>G	rs9648696	benign
*EGFR*	c.474C>T	rs2072454	benign
*EGFR*	c.560-84T>C	rs2075109	benign
*EGFR*	c.628+104C>T	rs2075110	benign
*EGFR*	c.629-62A>G	rs11506105	benign
*EGFR*	c.1006+151T>C	rs3735059	benign
*EGFR*	c.1562G>A	rs2227983	benign
**Mutations detected by both methods**			
*EGFR*	c.1881-600G>A	rs10228436	benign
*EGFR*	c.1887T>A	rs2227984	benign
*EGFR*	c.1920-215G>C	rs2241055	benign
*EGFR*	c.2283+96A>G	rs2017000	benign
*EGFR*	c.2284-60T>C	rs10241451	benign
*FBXW7*	c.1746G>A		
*NRAS*	c.-3343C>T		
*PIK3CA*	c.352+40A>G	rs3729674	benign
*PIK3CA*	c.1173A>G	rs2230461	benign
*PIK3CA*	c.2295-57C>G	rs2699889	benign
*PIK3CA*	c.*10365T>C		
*RNF43*	c.350G>A	rs2257205	benign
*TP53*	c.665+92T>G	rs12951053	benign
*TP53*	c.665+72C>T	rs12947788	benign
